# Newborn genetic screening of congenital adrenal hyperplasia using long-read sequencing

**DOI:** 10.1186/s13023-025-04116-1

**Published:** 2025-11-21

**Authors:** Yuqi Yang, Ying Wang, Bin Zhang, Bin Yu

**Affiliations:** Department of Medical Genetics, Changzhou Maternal and Child Health Care Hospital, Changzhou Medical Center of Nanjing Medical University, No.16 Ding Xiang Road, Changzhou, Jiangsu Province China

**Keywords:** Congenital adrenal hyperplasia, Long-read sequencing, Newborn genomic sequencing, Newborn screening, Single molecule sequencing

## Abstract

**Objective:**

To explore the use of genomic screening for congenital adrenal hyperplasia (CAH) based on long-read sequencing (LRS), aiming to provide an effective method for LRS-based screening (LRSBCS).

**Methods:**

All newborns underwent traditional CAH screening via the collection of dried blood spots. We conducted a retrospective clinical study of 73 cases, including 12 confirmed cases of CAH, 18 cases with false-positive biochemical screening results, and 43 healthy newborns as control. Full-length CAH-related genes, including *CYP21A2*, *CYP11B1*, *CYP17A1*, *HSD3B2*, and *STAR* were amplified and sequenced on a Sequel II platform (Pacific Biosciences).

**Results:**

Among the 235,999 newborns, 12 were confirmed to have CAH, based on biochemical and/or genetic testing. The positive-predictive values of the initial and positive recall results were 0.60% (12/1958) and 3.68% (12/326), respectively. The 12 children with CAH were accurately diagnosed using LRSBCS. For LRS, the Bayesian-estimated sensitivity is 96.2% (95% CrI: 80.3%–99.9%) and the specificity is 99.2% (95% CrI: 98.0%–99.9%). Eleven pathogenic variants of *CYP21A2* were detected, including eight SNVs/indels and three deletions. The most frequent variants were c.293–13 C > G (7/11) and c.518T > A (7/11). Furthermore, LRSBCS can directly report the characteristics of gene variants (cis or trans mutations) and effectively distinguish between functional genes and pseudogenes.

**Conclusions:**

LRSBCS represents a novel molecular screening approach tailored specifically for CAH, demonstrating preliminary feasibility in clinical settings.

**Clinical trial number:**

Not applicable.

**Supplementary Information:**

The online version contains supplementary material available at 10.1186/s13023-025-04116-1.

## Introduction

Congenital adrenal hyperplasia (CAH) is a group of genetically determined disorders characterised by multiple hormonal imbalances [1]. Most involve the androgen overproduction and variable deficiencies in cortisol and/or aldosterone, affecting metabolism and sexual development in infants, children, and adults. They can even be life-threatening [2]. CAH is a common autosomal recessive disease caused by mutations in genes encoding enzymes in the adrenal steroidogenic pathway, including steroid 21-hydroxylase (CYP21A2), 11b-hydroxylase (CYP11B1), 17a-hydroxylase (CYP17A1), and 3b-hydroxysteroid dehydrogenase type 2 (HSD3B2). Key pathogenic variants in genes associated with CAH include *CYP21A2*, *CYP11B1*, *CYP17A1*, *HSD3B2*, *STAR*, *POR*, *CYP11A1*, and *CYP11B2* [3, 4]. More than 90% of CAH cases are due to 21-hydroxylase-deficiency (21-OHD) caused by mutations in *CYP21A2*. This deficiency has three subtypes: classic salt wasting (SW), classic simple virilising (SV), and non-classic (NC-21OHD) subtypes.

Many studies have confirmed that newborn screening (NBS) for CAH enables early diagnosis and therapy, significantly improving the quality of life of affected children [5–8]. However, several problems hinder the widespread application of NBS. Firstly, current NBS can only detect classic 21-OHD by testing the levels of 17-hydroxyprogesterone (17α-OHP) in dried blood spots (DBS). Consequently, NC-21OHD and other CAH subtypes are often missed. Secondly, many factors influence 17α-OHP testing, such as DBS collection time, gestational age, birth weight, and testing method. Current screening methods, such as the dissociation-enhanced lanthanide fluoroimmunoassay (DELFIA) and liquid chromatography–tandem mass spectrometry (LC-MS/MS), are suboptimal, particularly due to their relatively high false-positive rate in CAH screening. The positive predictive value (PPV) ranges from 1.56% to 6.6% [9–11]. Thirdly, the low PPV can be improved by second-tier screening using DNA-based methods or LC-MS/MS [6]. However, its applications remain limited. Dynamic steroid metabolism testing by LC-MS/MS can still yield unreliable results in recall samples [12]. Therefore, a new CAH screening method is urgently needed to improve screening effectiveness.

With the rapid development of next-generation sequencing (NGS) in recent years, genetic testing has played a significant role in the screening and diagnosis of neonatal genetic diseases [13]. Newborn genome sequencing (nGS) is a technological innovation for NBS. Some studies, such as BabySeq [14], NBSeq [15], NC NEXUS [16], STATseq [17], NESTS [13], and Neoseq [18], have confirmed that nGS can further expand the screening for neonatal diseases, provide more detailed genetic information, help clinicians make timely diagnoses, and formulate personalised treatment plans. However, due to the specificity of the pathogenic genes, nGS may have limited efficacy for CAH screening. The 21-OH encoding gene *CYP21A2* has a highly homologous pseudogene (98%), named *CYP21A1P*. This limits the accurate identification of *CYP21A2* by NGS due to pseudogene disturbances. Wang et al. used targeted gene panel sequencing for the molecular diagnosis of CAH and reported a diagnostic rate of 98.5% [19]. However, that study focused on diagnosis rather than screening. The authors also stated that detection of structural variations in *CYP21A2* using NGS was limited. Single-molecule real-time (SMRT) long-read sequencing (LRS) offers several advantages, particularly obtaining much longer sequencing reads. This contributes to the discrimination of highly homologous genes. Liu et al. [20] successfully established an effective screening and diagnosis method for CAH based on LRS. Li et al. [21] reported a full sequence analysis of *CYP21A2* and *CYP21A1P* through long-range locus-specific polymerase chain reaction (PCR) followed by LRS and proposed that LRS was comprehensive and intuitive for results interpretation.

We aimed to provide an effective method for the genomic screening of newborns with CAH. In this study, we initiated a new project for nGS based on LRS. We explored the clinical application of LRS-based CAH screening (LRSBCS), and compared its efficacy in different newborn populations.

## Materials and methods

### Study design and participants

This retrospective study was conducted at the Department of Medical Genetics, Changzhou Maternal and Child Health Care Hospital. The research involving human participants complied with national regulations, institutional policies, and the tenets of the Helsinki Declaration, and has been approved by the Ethics Committee of Changzhou Maternal and Child Health Care Hospital (2023-3).

From January 2017 to July 2023, all neonates born at the Changzhou Maternal and Child Health Care Hospital were enrolled in the NBS program. All subjects underwent traditional CAH screening via collection of DBS. Seventy-three newborns from the NBS program were selected as participants, including 12 cases confirmed to have CAH, 18 cases with false-positive biochemical screening results, and 43 healthy newborns (negative NBS screening and normal growth and development after follow-up). The newborns’ parents had written informed consent to publish this information.

### Sample collection

DBS were collected onto 903 filter paper (Wallace Oy, Turku, Finland) from all infants within 72 h after birth. For experimental validation, peripheral blood samples were collected from parents of positive-testing newborns.

### Traditional biochemical screening of CAH

Based on the importance of early diagnosis and intervention for positive-testing infants, we simultaneously adopted a strategy for diagnosis and treatment. Traditional biochemical screening (TBS) for CAH was performed using a time-resolved fluoroimmunoassay (TRFIA). Newborn 17α-OHP levels were detected using TRFIA with a Wallac 1235 Auto DELFIA (Perkin Elmer, Waltham, MA, USA). If the initial screening result was positive (after reanalysis of the original sample), the infant was recalled. For cases testing positive after recall, an adjunct examination was performed, mainly including routine blood tests, assessment of liver and kidney function, and hormone tests. The target gene *CYP21A2* is detected using Sanger sequencing and/or multiplex ligation-dependent probe amplification (MLPA) techniques. The scope of the detection encompasses the proband and their parents, aiming to validate the suspected variant loci., please refer to the previous reports [22, 23].

### Newborn genetic screening of CAH by LRS

*LRS of CAH-related genes*. Full-length CAH-related genes, including *CYP21A2*, *CYP11B1*, *CYP17A1*, *HSD3B2*, and *STAR*, were amplified as previously described [20]. Multiplex long-range PCR, using KOD FX Neo (Toyobo, Osaka, Japan) was employed to achieve optimal elongation rates and capabilities using genomic DNA as template. PCR products were confirmed by agarose gel electrophoresis, purified with 1× Ampure PB beads (Pacific Biosciences, Menlo Park, CA, USA), and quantified using a Qubit dsDNA BR assay kit (Thermo Fisher Scientific, Waltham, MA, USA). Pre-libraries for single-molecule real-time (SMRT) sequencing by Pacific Biosciences were constructed using a one-step end-repair and ligation reaction to add a unique barcode adaptor (Integrated DNA Technologies, Coralville, IA, USA). The barcoded pre-libraries were pooled, purified with 0.6× Ampure PB beads, and quantified using a Qubit dsDNA HS assay kit. The Sequel Binding Kit (version 2.0) and Internal Control Kit (version 1.0) (Pacific Biosciences) were used to generate the SMRTbell library. The SMRT-link Sample Setup module was employed for primer annealing and polymerase binding in the SMRT cells, and sequencing was performed on the Sequel II platform (Pacific Biosciences) with the Sequel II Sequencing Kit 2.0. High-quality circular consensus sequencing (CCS) reads were obtained from raw subreads (BAM files) with an accuracy ≥ Q20 and were split into individual samples based on barcodes using the SMRT-Link analysis software suite (Pacific Biosciences). The mean sequencing depth in this study was 1,214× (range: 65×–3,731×). The minimum coverage depth threshold was set at 30×. The filtered data were then aligned to the human GRCh38/hg38 genome sequence using pbmn2 for the target genes (*CYP11B1*,* HSD3B*,* CYP17A1*,* and STAR*). Single-nucleotide variations (SNVs) and small insertions and deletions (indels) were identified using FreeBayes 1.3.4 (https://github.com/freebayes/freebayes/releases), and haplotypes were analyzed using Whatshap. For the analysis of *CYP21A2* and *CYP21A1P*, reads containing specific primer pairs as previously mentioned were assigned as pseudogene *CYP21A1P*, functional gene *CYP21A2*, 30-kb deletion *CYP21A1P/CYP21A2* chimera, and large gene conversion *CYP21A2/CYP21A1P* chimera [20]. These reads were aligned to the CYP21A2-TNXB reference for SNVs and indels analysis. The junction regions of the chimera reads were identified using signature SNVs and indels derived from the alignment between the CYP21A2-TNXB and CYP21A1P-TNXA references [20]. Pathogenic variants were visualized in the Integrative Genomics Viewer(IGV) program (https://igv.org/doc/desktop/) with BAM files.

*Data analysis of long-read sequencing.* High-quality circular consensus sequencing (CCS) reads were obtained from raw subreads (BAM files), split into individual samples based on barcodes, and aligned to human GRCh38/hg38 genome sequences using the SMRT-link analysis software suite (Pacific Biosciences). FreeBayes 1.3.4 (https://github.com/freebayes/freebayes/releases) was employed to identify single-nucleotide variations (SNVs) and small insertions and deletions (indels). To analyse 30-kb deletions in *CYP21A2* and *CYP21A1P*, the *CYP21A1P*/*CYP21A2* chimera reads were used to show the SNVs/indels and junction regions with the Integrative Genomics Viewer (https://igv.org/doc/desktop/). The *CYP21A2*, *CYP21A1P*/*CYP21A2*, and *CYP21A2*/*CYP21A1P* chimera reads were aligned to the reference build *CYP21A2-TNXB* for analysis of SNVs and indels. The junction region of the chimera reads was identified by the signature SNVs and indels obtained from the alignment between the reference builds *CYP21A2-TNXB* and *CYP21A1P-TNXA*.

*Scoring Criteria for Variant Site Interpretation*: Variant interpretation has been performed according to the guidelines from ACMG (American College of Medical Genetics and Genomics) [24]. The pathogenicity of the variants was evaluated using at least frequency checks in populations (dbSNP, 1000 Genomes, ExAC), searches in various databases (HGMD, HGVS, ClinVar), and in silico analyses (PolyPhen-2, SpliceAI) [25]. Detailed ACMG classification and evidence levels for variants were provided in Supplementary Table 1. In order to validate the pathogenic and likely pathogenic variants identified by LRS, Sanger sequencing validation was conducted separately for Case 1, Case 3, and Case 10.

## Results

In total, 235,999 newborns underwent TBS for CAH, including 123,101 males and 112,898 females. In total, the initial results were positive in 1,985 infants, and their 17α-OHP levels exceeded the reference range (12 nmol/L). The positivity rate for the initial screening was 0.84% (1,985/235,999). After recall, 1,939 infants underwent a second DBS test. The recall rate after the initial screening was 97.68% (1,939/1,985). The 17α-OHP levels of 326 recalled cases exceeded 12.0 nmol/L. The positivity rate in the recalled patients was 16.81% (326/1,939). CAH was diagnosed in 12 infants (8 males, 4 females) based on biochemical and/or genetic testing. The incidence of CAH was 1:19,667 (1:15,388 for males and 1:28,225 for females). The PPVs of the initial and positive recall results were0.60%(12/1985) and 3.68% (12/326), respectively.

**Fig. 1:**
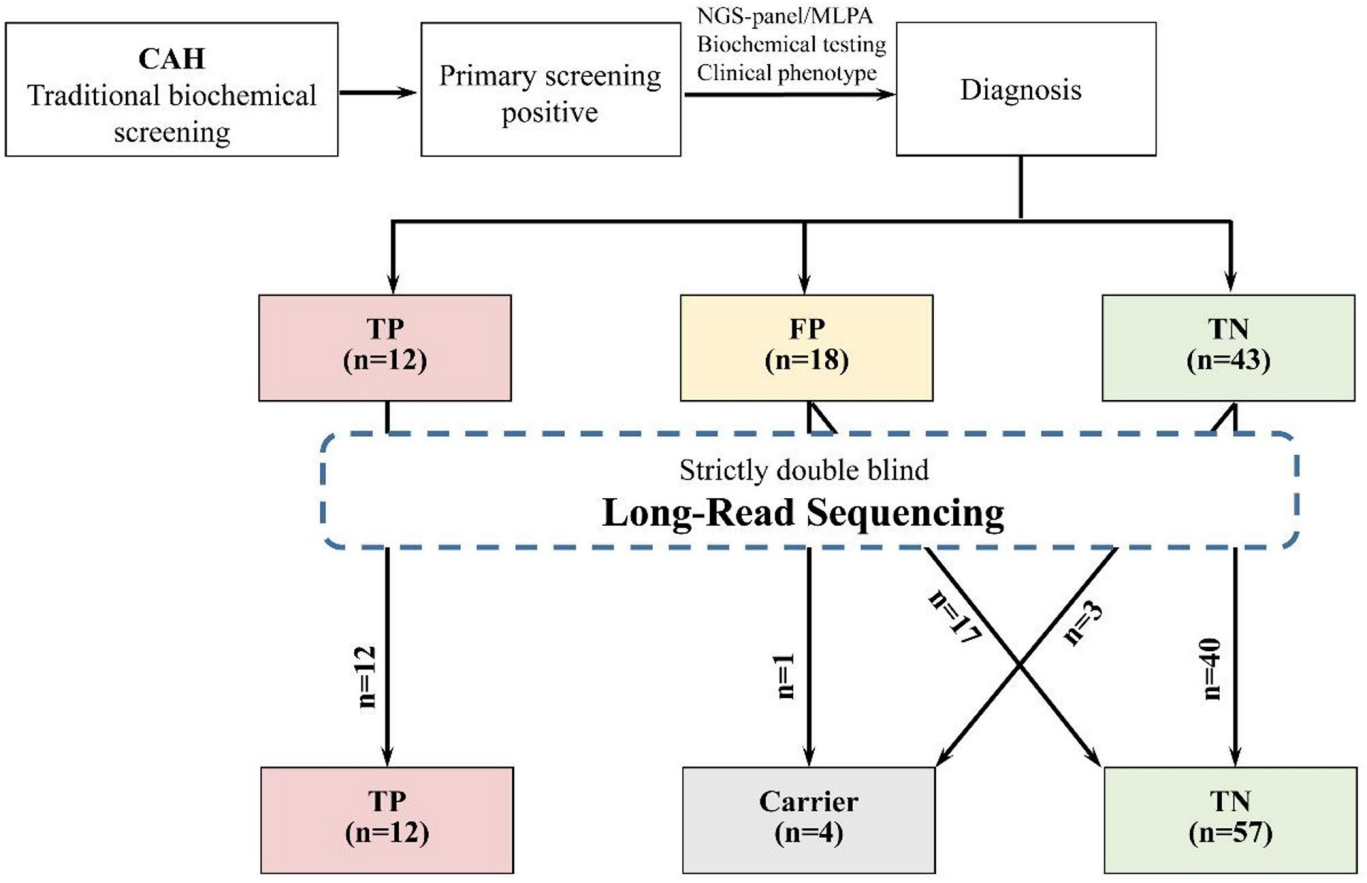
The flow chart and result overview of this study. Note: TBS: Traditional biochemical screening TP: True positive FP: False positive TN: True negative

LRSBCS was performed in 73 infants, including confirmed CAH cases and false-positive/false-negative cases (Fig. 1). Overall, both LRSBCS and TBS identified all 12 confirmed cases (100%). In addition, 18 false-positive and 43 true-negative cases were accurately identified using LRSBCS, and four carriers were identified (Supplementary Table 2). The false-positive rate was 0. Sensitivity and specificity(TP = 12,TN = 61,FP = 0,FN = 0)were estimated through Bayesian analysis employing the Jeffreys non-informative prior (Beta[0.5, 0.5]). The analysis yielded a posterior sensitivity estimate of approximately 96.2% (95% credible interval [CrI]: 80.3%–99.9%) and a specificity estimate of around 99.2% (95% CrI: 80.3%–99.9%), with corresponding posterior distributions modeled as Beta(12.5, 0.5) and Beta(61.5, 0.5), respectively. The narrow credible interval for specificity reflects high confidence in negative case detection, whereas the broader interval for sensitivity is attributable to the limited number of positive cases, a pattern well-supported by statistical principles. These results demonstrate the high diagnostic performance of the method for the condition under investigation.

**Fig. 2:**
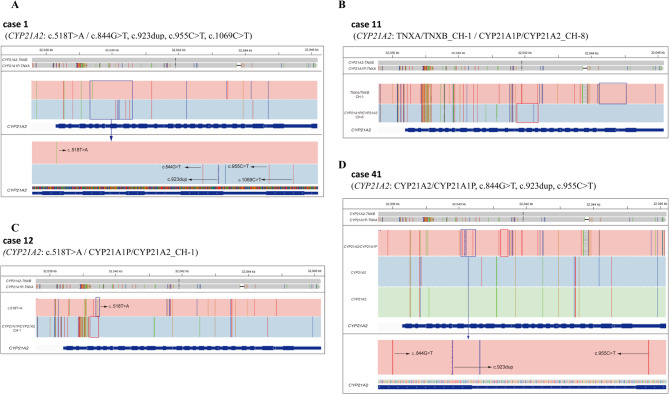
IGV plots of LRS results showing variations of CAH genes. Note **A**. Five pathogenic variants were detected in case 1. **B**. The deletion of exon 1 ~ 7 of *CYP21A2* gene was detected in case 11. **C**. The pathogenic variant c.518T > A and the deletion of CYP21A1P/CYP21A2_CH-1 was detected in case 12. **D**. The fusion of real gene and pseudo gene of *CYP21A2* gene was detected in case 41

Table 1 shows the results of traditional NBS and the LRSBCS in 12 children with CAH. First, after being recalled due to the positive results on initial screening of 17α-OHP, nine cases were found to carry pathogenic variants in *CYP21A2* by Sanger sequencing and/or MLPA, and were also detected by LRSBCS. In addition, LRSCS detected pathogenic variants in all 12 cases, including 10 cases with SNVs, one case with a deletion, and one case with compound heterozygosity. Four of the 12 cases were homozygous for pathogenic variants and eight were heterozygous. Second, as shown in Tables 2 and 11 types of pathogenic variants were detected in this study, including eight SNVs/indels and three deletions. The most frequent variants were c.293–13 C > G (7/11) and c.518T > A (7/11). Third, using the TBS method, Sanger sequencing detected variants of *CYP21A2*, after which we tested the blood of the parents by Sanger sequencing to determine the origin of the variants in the offspring. Moreover, LRSBCS could directly report the characteristics of gene variants (cis or trans mutations). For example, we identified five pathogenic variants in Case 1 by LRS: c.518T > A, c.844G > T, c.923dup, c.955 C > T, and c.1069 C > T. By analysis of sequencing data, we could directly confirm that c.518T > A was in trans with other variants. However, we could only determine the origin using Sanger sequencing of additional parental blood samples in TBS. In fact, c.518T > A originated from the patient’s father, while all other variants originated from her mother (Fig. 2A).

**Table 1:** Comparison of LRS and traditional biochemical screening in 12 CAH children

Case	Age	Gender	Traditional biochemical screening			Follow up	Long-Read Sequencing			
			Initial screening value (17α-OHP, nmol/L)	Sanger/MLPA	Origin		Affected gene	Genotype	Type	Variant
1	3Y	Female	133	c.518T > A	f	NA	*CYP21A2*	c.518T > A(SV)^t^	P	CHet
				c.844G > T	m			c.844G > T(NC)	P	
				c.923_924insT	m			c.923dup(SW)	P	
				c.955 C > T	m			c.955 C > T(SW)	P	
				c.1069 C > T	m			c.1069 C > T(SW)	P	
2	2Y	Male	52.8	c.293–13 A/C > G	f	NA	*CYP21A2*	c.293–13 C > G(SW)	P	CHet
				c.518T > A, p.(ILe173Asn)	m			c.518T > A(SV) ^t^	P	
								c.10712G > A	VUS	
							*STAR*	c.179-14G > A	VUS	
3	2Y	Male	706	c.293–13 A/C > G	f	Death	*CYP21A2*	c.293–13 C > G(SW) ^t^	P	CHet
				c.740delA, p.(Glu247Glyfs*11)	m			c.740del(SW)	P	
								c.*1316C > T	VUS	
4	2Y	Male	64.5	c.293–13 A/C > G	m	NA	*CYP21A2*	c.293–13 C > G(SW) ^t^	P	CHet
				c.518T > A, p.(ILe173Asn)	f			c.518T > A(SV)	P	
5	8 M	Male	44.9	--	--	NA	*CYP21A2*	c.293–13 C > G(SW)	P	CHet
								c.518T > A(SV) ^t^	P	
								c.*1215C > T	VUS	
								c.*1316C > T	VUS	
								c.*1351G > C	LP	
6	4 M	Male	604	--	--	NA	*CYP21A2*	c.293–13 C > G(SW)	P	hom
7	4Y	Male	102	c.293–13 C>G	m	NA	*CYP21A2*	c.293–13 C > G(SW)	P	CHet
				c.518T>A	f			c.518T > A(SV) ^t^	P	
8	5Y	Male	612	c.293–13 C>G	f m	NA	*CYP21A2*	c.293–13 C > G(SW)	P	hom
9	8Y	Male	159	c.518T>A	f m	NA	*CYP21A2*	c.518T > A(SV)	P	hom
10	2Y	Female	29.8	c.92 C > T	f m	NA	*CYP21A2*	c.92 C > T	P	hom
11	7Y	Female	527	*CYP21A2* Exon 1-7del	f m	NA	*CYP21A2*	TNXA/TNXB_CH-1(SW)	P	CHet
								CYP21A1P/CYP21A2_CH-8(SW) ^t^	P	
12	5Y	Female	20.1 (1030 for recall)	--	--	NA	*CYP21A2*	c.518T > A(SV)	P	CHet
								CYP21A1P/CYP21A2_CH-1(SW) ^t^	P	

Note:

Y: year. M: Month

f: father. m: mother

SW: salt-wasting. SV: simple virilizing. NC: non-classical

P: Pathogenic. LP: Likely pathogenic. VUS: variant of uncertain significance

CHet: compound heterozygous. hom: homozygous

NA: No abnormalities of physical development during the treatment

t: Trans-mutation with others

MLPA: multiplex ligation-dependent probe amplification

The reference range of 17α-OHP is 0-12nmol/L

**Table 2:** The pathogenic variants in this study

Gene	Type of variation	Nucleotide variation	Allele number	Phenotype	Junction region
*CYP21A2*	SNV/indels	c.293–13 C > G	7/11	SW	chr6:320 39,081
	SNV/indels	c.518T > A	7/11	SV	chr6:320 39,426
	SNV/indels	c.1069 C > T	1/11	SW	chr6:320 40,535
	SNV/indels	c.740del	1/11	SW	chr6:320 40,005
	SNV/indels	c.844G > T	1/11	NC	chr6:320 40,110
	SNV/indels	c.923dup	1/11	SW	chr6:320 40,182
	SNV/indels	c.92 C > T	1/11	--	chr6:320 38,514
	SNV/indels	c.955 C > T	1/11	SW	chr6:320 40,421
	30-kb deletions	CYP21A1P/CYP21A2_CH-1	1/11	SW	-
	30-kb deletions	CYP21A1P/CYP21A2_CH-8	1/11	SW	
	30-kb deletions	TNXA/TNXB_CH1	1/11	SW	-

Note:

SW: salt-wasting

SV: simple virilizing

NC: non-classical

SNV: single nucleotide variation

Case 11 is a representative example highlighting the advantages of LRS. The girl was recalled due to her initial screening value (17α-OHP) was 527 nmol/L. Sanger sequencing did not detect any variations in the disease-causing genes. However, the deletion of exons 1–7 of *CYP21A2* was reported by multiplex ligation-dependent probe amplification (MLPA). However, deletion heterozygosity was accurately detected using LRSBCS (Fig. 2B).

Notably, Case 12 was overlooked during the biochemical screening, yet was ultimately confirmed as a compound heterozygote via LRSBCS. The girl underwent 17α-OHP testing on postnatal day 4, yielding a result of 20.1 nmol/L. Owing to the family’s preference, no secondary screening was conducted for this parameter. However, she returned to the hospital due to an external genital malformation after half a month. After re-examining the DBS, the level of 17α-OHP was found to reach 1,030 nmol/L, and was diagnosed as delayed CAH, according to the clinical phenotype. In the present study, the pathogenic variant c.518T > A and the deletion of CYP21A1P/CYP21A2_CH-1 in *CYP21A2* were detected using LRS (Fig. 2C).

Four carriers were identified by LRSBCS (Cases 30, 41, 59, and 60; Supplementary Table 1). Notably, three of these cases were associated with the fusion of functional and pseudogenes. For example, although three pathogenic variants of *CYP21A2* were detected in case 41, two copies of the real gene remained. One copy was a duplication caused by fusion of the real and pseudogenes (Fig. 2D). Therefore, these children were predicted to be healthy. LRS technology can thus effectively distinguish between functional genes and pseudogenes. The Sanger sequencing results for pathogenic and likely pathogenic loci can be found in Supplementary Fig. 1.

Moreover, screening data of 235,999 people in this area showed that the PPV was only 3.68%. In this retrospective cohort study, no false-positive results were obtained for any of the 73 samples after LRSBCS, with a false-positive rate of 0. Simultaneously, 18 false-positive cases from TBS were reported as negative.

## Discussion

In this study, PPVs for the initial and positive recall results were 0.60% (12/1958)and 3.68% (12/326), respectively. Moreover, 12 children with CAH were accurately diagnosed using LRSBCS.

Irrespective of whether TBS or tandem mass spectrometry screening is used, NBS undoubtedly plays a major role in the early detection, treatment, and improved outcomes of children with CAH. However, some of the limitations of current NBS cannot be ignored. These primarily involve two aspects. First, some false negatives are obtained when using the current screening methods owing to technical limitations and the complexity of CAH types. TBS is based on detecting the level of 17α-OHP in DBS, which identifies about 70% of classic 21-OHD cases. However, non-classic 21-OHD and other types of CAH are frequently missed by current screening [26]. In fact, diagnosis of case 12 was missed in the initial screening. Fortunately, this patient ultimately had their etiological factor identified through genetic screening and subsequently received treatment. However, the treatment was delayed for more than a month. Second, factors like premature delivery and emergency responses contribute to low specificity and a high false-positive rate in CAH screening, resulting in unnecessary recalls. For example, our data from 235,999 people in Changzhou City showed that the PPV among recalled positives was only 3.68%. Some studies reported that the PPV was approximately 1.5–6.6% globally [9–11]. Therefore, technological innovations are needed to improve CAH screening.

In this study, we developed a new method for the molecular screening of CAH, named LRSBCS. Although this was a small-sample retrospective study, it clearly demonstrated the advantages of single-molecule real-time long-read sequencing. First, the effect of CAH molecular screening based on the LRS was satisfactory, with both detection rate and specificity at 100%. The LRSBCS design included five common CAH-related pathogenic genes (*CYP21A2*, *CYP11B1*, *CYP17A1*, *HSD3B2*, and *STAR*). Theoretically, all types of pathogenic variations can be detected at the same time in one test; these include point mutations, small deletions, insertions, large fragment deletions/duplications, and gene conversions. Although only *CYP21A2* variants were found in the positive cases, LRSBCS accurately distinguished the variant types. In addition, LRSBCS can directly analyse the cis–trans relationship between pathogenic variants and determine their origin without relying on family genotyping. This capability is superior to traditional genetic analysis methods. Moreover, the LRSBCS accurately distinguished *CYP21A2* and *CYP21A1P*. The results are intuitive and unaffected by the fusion of real and pseudo-genes. Importantly, *CYP21A2* has a highly homologous pseudogene *CYP21A1P*. The homology of their exon sequences is 98% and that of introns is 96%, making it difficult to analyse CAH genotypes using traditional genetic techniques. In particular, NGS cannot distinguish the characteristics of fragments, leading to unreliable results.

Approximately 90%–95% of 21-OHD cases are related to variations in *CYP21A2*. The 12 children with CAH in this study all had pathogenic variants in this gene. Consistent with some studies [19, 27], point mutations of *CYP21A2* were the predominant variant type mode of variation in children with CAH. c.293–13 C >G (7/11) and c.518T >A (7/11) were the most frequent variants. Furthermore, LRSBCS can reduce the false-positive results of TBS. Owing to the influence of blood collection time, gestational age, birth weight, and experimental method, the high false-positive rate and low PPV of CAH screening remain problematic, particularly for some special populations, such as premature infants [28]. The high false-positive rate leads to unnecessary recall of numerous healthy infants, which will not only burden the medical system, but will also impose a psychological burden on the parents [29]. In this study, 18 false-positive cases of traditional CAH screening were reported to be negative by LRSBCS, while no false-positive cases were detected.

Nonetheless, this study has limitations. This was a retrospective study with a small sample size, which could not fully reflect the screening process. We also did not compare the pivotal factors of screening methods, such as experimental time and cost. Therefore, the advantages of LRSBCS require further elucidation. Large-scale and multicentre prospective studies are needed to confirm its advantages. The LRSBCS design included five common CAH-related pathogenic genes. Due to the small sample size, only pathogenic variations in *CYP21A2* were detected in this study. More clinical samples are required to confirm the screening effectiveness of LRSBCS for other types of CAH. Due to CAH’s high genetic heterogeneity, this sequencing protocol is better suited for screening neonates and asymptomatic individuals, but has limited utility in confirmatory genetic diagnosis.

As previously reported, 21-Hydroxylase deficiency (21-OHD) is the most common form of CAH, accounting for more than 95% of all cases with congenital adrenal hyperplasia (CAH) [1]. The second most common form of CAH is 11β-hydroxylase deficiency (11β-OHD), which is caused by mutations in the *CYP11B1* gene and accounts for 5–8% of all CAH cases [4]. 17ɑ-hydroxylase deficiency (17OHD) caused by pathogenic variants in the *CYP17A1* gene is a rare form of CAH which accounts for about 1% of all CAH cases. Other forms caused by deficiency of 3β-hydroxysteroid dehydrogenase type 2 (HSD3B2), P450 cholesterol side-chain cleavage enzyme (CYP11A1), cytochrome P450 oxidoreductase (POR), or the steroidogenic acute regulatory protein (STAR) are very or extremely rare. The panel applied in this study covered a total of five CAH-related genes (*CYP21A2*,* CYP11B1*,* CYP17A1*,* HSD3B2*, and *STAR*) and represents a novel molecular screening approach with 100% detection rate and specificity. However, other rarer forms, including those caused by mutations of *CYP11A1* and *POR* as well as those relevant to rarer CAH subtypes, are omitted, leading to the risk of false negatives. The large size of the *CYP11A1* (29.9 kb) and *POR* (71.7 kb) genes and the associated twofold higher cost limited their inclusion in the current panel. With the decreasing cost of LRS, this limitation could justify expanding the panel in future designs.

In conclusion, LRSBCS represents a novel molecular screening approach tailored specifically for CAH, demonstrating preliminary feasibility in clinical settings. This approach can detect all types of pathogenic variants by one test, directly determine the origin of variation without relying on family analysis, is not hampered by the homology of functional and pseudogenes, and significantly reduces the false-positive rate of TBS.

## Supplementary Information

Below is the link to the electronic supplementary material.

Supplementary Material 1

Supplementary Material 2

Supplementary Material 3

## Data Availability

The questionnaire and datasets used are available from the corresponding author on request.

## Abbreviations

CAH: Congenital adrenal hyperplasia
LRS: Long-read sequencing
SMRT: Single-molecule real-time
LRSBCS: LRS-based screening
NBS: Newborn screening
17α-OHP: 17-hydroxyprogesterone
DBS: Dried blood spots
LC-MS/MS: Liquid chromatography–tandem mass spectrometry
PPV: Positive predictive value
NGS: Next-generation sequencing
nGS: Newborn genome sequencing
