# Supplementary figures and images for: Newborn genetic screening of congenital adrenal hyperplasia using long-read sequencing
Source: Orphanet J Rare Dis. 2025 Nov 21;20:599. doi: 10.1186/s13023-025-04116-1 (PMC12639871; doi:10.1186/s13023-025-04116-1)

**Figure S1****A Case 1 (*CYP21A2*: c.518T>A/c.844G>T; c.923dupT; c.955C>T; c.1069C>T)**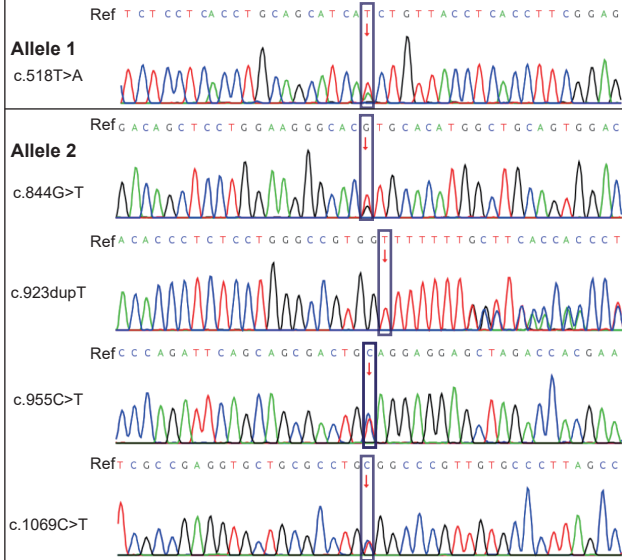**B Case 3 (*CYP21A2*: c.293-13C>G/c.740delA)**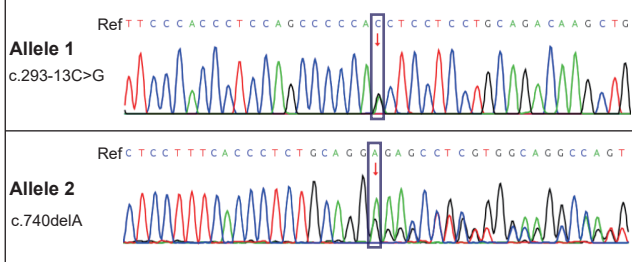**C Case 10 (*CYP21A2*: c.92C>T Homo)**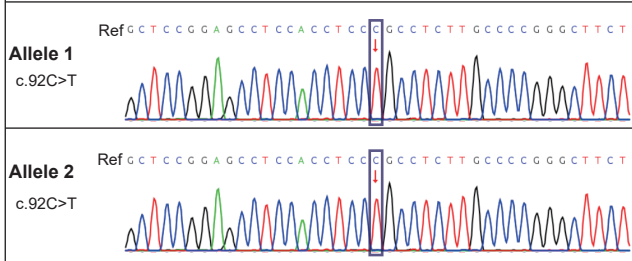

Supplement: Supplementary file 1 — Supplementary Material 1 [file 13023_2025_4116_MOESM1_ESM.pdf]
